# Tackling amyloidogenesis in Alzheimer’s disease with A2V variants of Amyloid-β

**DOI:** 10.1038/srep20949

**Published:** 2016-02-11

**Authors:** Giuseppe Di Fede, Marcella Catania, Emanuela Maderna, Michela Morbin, Fabio Moda, Laura Colombo, Alessandro Rossi, Alfredo Cagnotto, Tommaso Virgilio, Luisa Palamara, Margherita Ruggerone, Giorgio Giaccone, Ilaria Campagnani, Massimo Costanza, Rosetta Pedotti, Matteo Salvalaglio, Mario Salmona, Fabrizio Tagliavini

**Affiliations:** 1 Neurology V and Neuropathology Unit, IRCCS Foundation “Carlo Besta” Neurological Institute (INCB), Via Celoria 11, 20133 Milan, Italy; 2 Neuroimmunology and Neuromuscular Disorder Unit, IRCCS Foundation “C. Besta” Neurological Institute (INCB), Via Celoria 11, 20133 Milan, Italy; 3 Department of Molecular Biochemistry and Pharmacology, IRCCS-Istituto di Ricerche Farmacologiche “Mario Negri”, Via La Masa 19, 20158 Milan, Italy; 4 Department of Chemical Engineering, University College London, London WC1E 7JE, UK

## Abstract

We developed a novel therapeutic strategy for Alzheimer’s disease (AD) exploiting the properties of a natural variant of Amyloid-β (Aβ) carrying the A2V substitution, which protects heterozygous carriers from AD by its ability to interact with wild-type Aβ, hindering conformational changes and assembly thereof. As prototypic compound we designed a six-mer mutated peptide (Aβ1-6_A2V_), linked to the HIV-related TAT protein, which is widely used for brain delivery and cell membrane penetration of drugs. The resulting molecule [Aβ1-6_A2V_TAT(D)] revealed strong anti-amyloidogenic effects *in vitro* and protected human neuroblastoma cells from Aβ toxicity. Preclinical studies in AD mouse models showed that short-term treatment with Aβ1-6_A2V_TAT(D) inhibits Aβ aggregation and cerebral amyloid deposition, but a long treatment schedule unexpectedly increases amyloid burden, although preventing cognitive deterioration. Our data support the view that the Aβ_A2V_-based strategy can be successfully used for the development of treatments for AD, as suggested by the natural protection against the disease in human A2V heterozygous carriers. The undesirable outcome of the prolonged treatment with Aβ1-6_A2V_TAT(D) was likely due to the TAT intrinsic attitude to increase Aβ production, avidly bind amyloid and boost its seeding activity, warning against the use of the TAT carrier in the design of AD therapeutics.

Alzheimer’s disease (AD) is the most common form of dementia in the elderly. Its clinical course is slow but irreversible since no disease-modifying treatments are currently available. As a result, this illness has a huge socio-sanitary impact and designing of effective therapies is considered a public health priority.

A central pathological feature of AD is the accumulation of misfolded Amyloid-beta (Aβ) peptides in the form of oligomers and amyloid fibrils in the brain^1^,^2^,^3^. It has been advanced that aggregated Aβ species, particularly oligomeric assemblies, trigger a cascade of events that lead to hyperphosphorylation, misfolding and assembly of the tau protein with formation of neurofibrillary tangles and disruption of the neuronal cytoskeleton, widespread synaptic loss and neurodegeneration. According to this view, altered Aβ species are the primary cause of AD and the primary target for therapeutic intervention^3^,^4^.

Aβ peptides derive from proteolytic processing of a large (695/770 amino acids) type 1 transmembrane glycoprotein known as amyloid beta precursor protein (APP). APP is cleaved at the N-terminus of the Aβ domain by β-secretase, forming a large, soluble ectodomain (sAPPβ) and a 99-residue, membrane-retained C-terminal fragment (C99). Subsequently, γ-secretase cleaves C99 to release Aβ with different carboxyl termini, including Aβ40, Aβ42 and other minor species^5^. APP may undergo an alternative, non-amyloidogenic processing where the protein is cleaved within the Aβ domain by α-secretase, forming a soluble ectodomain (sAPPα) and an 83-residue C-terminal fragment (C83)^5^,^6^.

We identified a novel mutation in the APP gene resulting in A-to-V substitution at codon 673, corresponding to position 2 in the Aβ sequence^7^. Studies on biological samples from an A673V homozygous carrier, and cellular and *C. elegans* models indicated that this mutation shifts APP processing towards the amyloidogenic pathway with increased production of amyloidogenic peptides. Furthermore, the A2V substitution in the Aβ sequence (Aβ_A2V_) increases the propensity of the full-length peptides (i.e., Aβ1-40 and Aβ1-42) to adopt a β-sheet structure, boosts the formation of oligomers both *in vitro* and *in vivo* and enhances their neurotoxicity^8^,^9^,^10^. Following the observation that humans carrying the mutation in the heterozygous state do not develop AD, we carried out *in vitro* studies with synthetic peptides that revealed the extraordinary ability of Aβ_A2V_ to interact with wild-type Aβ (Aβ_WT_), interfering with its nucleation or nucleation-dependent polymerization^7^. This provides grounds for developing a disease-modifying therapy for AD based on modified Aβ_A2V_ peptides retaining the key functional properties of parental full-length Aβ_A2V_.

Following this approach, we generated a mutated six-mer peptide (Aβ1-6_A2V_), constructed entirely by D-amino acids [Aβ1-6_A2V_(D)] to increase its stability *in vivo*, whose interaction with full-length Aβ_WT_ hinders oligomer production and prevents amyloid fibril formation^8^.

These results prompted us to develop a prototypic compound by linking Aβ1-6_A2V_(D) to an all-D form of TAT sequence [TAT(D)], a peptide derived from HIV that powerfully increases virus transmission to neighbour cells^11^, and is widely used for brain delivery of drugs^12^,^13^,^14^. Here we report that this compound [Aβ1-6_A2V_TAT(D)] has strong anti-amyloidogenic effects *in vitro*, leading to inhibition of oligomer, amyloid fibril formation and of Aβ-dependent neurotoxicity. Preclinical studies showed that a short-term treatment with this peptide in an AD mouse model prevents Aβ aggregation and amyloid deposition in the brain but longer treatment unexpectedly increases amyloid burden, most likely due to the TAT intrinsic attitude to enhance Aβ production and to avidly bind amyloid and boost its seeding activity, warning against the use of this carrier in therapeutic approaches for AD.

## Results

### In silico molecular modeling of Aβ_A2V_ peptide variants

**Figure 1:**
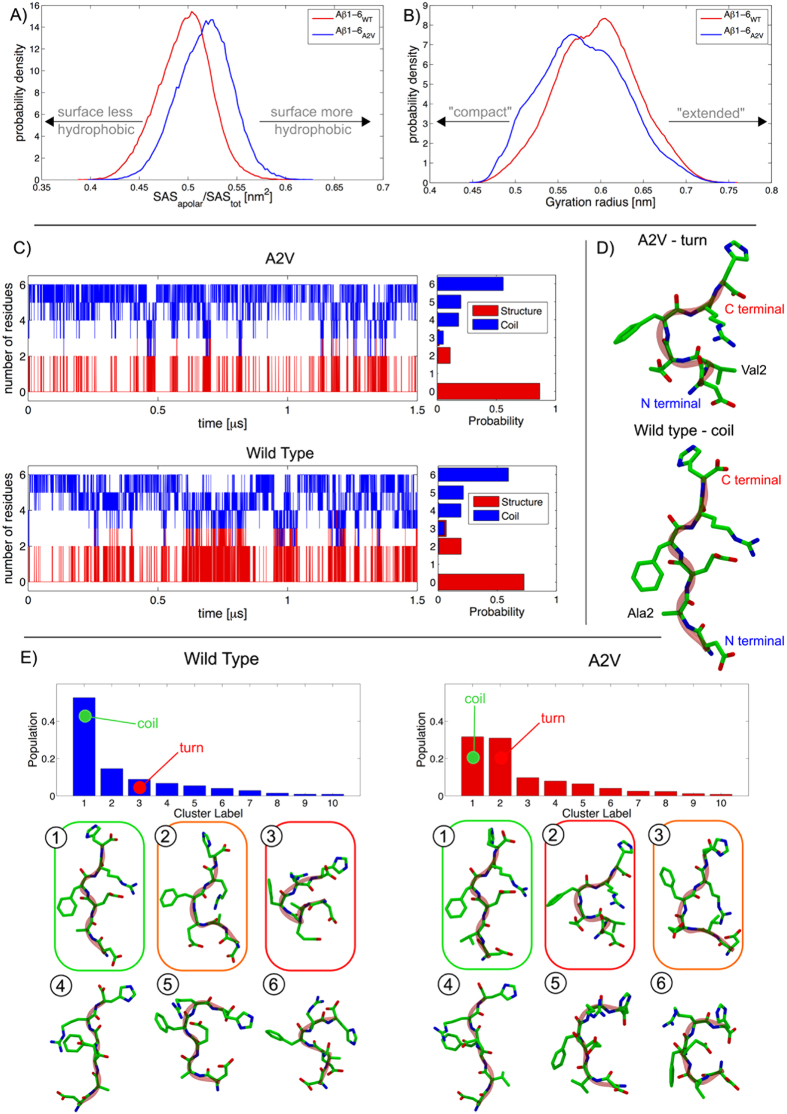
Analysis of 1.5 μs explicit solvent MD simulations of the Aβ1-6_WT_ and Aβ1-6_A2V_ peptides. (**A**) Apolar character of the peptide SAS represented as the ratio between SAS_apolar_ and the total SAS. (**B**) Gyration radius distribution. (**C**) Analysis of secondary structure propensity. “Structure” indicates residues possessing a defined secondary structure, in this case structure indicates residues in a “turn” configuration. “Coil” indicates residues that do not display a defined secondary structure. Analysis of the secondary structure was carried out with DSSP. (**D**) Typical compact “turn” and elongated “coil” configurations reported for the Aβ1-6_A2V_ and Aβ1-6_WT_, respectively. (**E**) Analysis of the most populated structural clusters. Representative structures of the six most probable clusters were reported. The coil configuration has been highlighted in green, the turn in red and a partly folded turn in orange.

To predict the structural basis of the anti-amyloidogenic effect of Aβ1-6_A2V_(D), a comparative conformation analysis of WT and mutated Aβ1-6 was carried out with all-atom classical MD simulations in explicit solvent. Both Aβ1-6_WT_ and Aβ1-6_A2V_ are intrinsically disordered peptides characterized by high flexibility. Nevertheless, the substitution of Ala2 with a Val residue induces significant changes in the appearance of the peptide in solution, resulting in an increase of the apolar character of the solvent accessible surface (SAS) (Fig. 1A) and in a modification of the gyration radius distribution in the Aβ1-6_A2V_. Figure 1B shows that the probability distribution of the gyration radius is characterized by a global shift to smaller values and by the appearance of a shoulder in the distribution corresponding to gyration radii of 0.5 nm.

An analysis of the secondary structure content displayed by the peptides (Fig. 1C) shows that, while both Aβ1-6_A2V_ and Aβ1-6_WT_ display a predominant coil configuration, Aβ1-6_A2V_ shows a slightly higher propensity to form secondary structure motifs involving two to three residues. Aβ1-6_A2V_ in fact displays a propensity to form a turn involving the Glu3, Phe4 and Arg5 residues (Fig. 1D). The most populated structural clusters^15^ (Fig. 1E), in Aβ1-6_WT_ are characterized by an elongated coil structure accounting for 52.6% of the configurations, while the compact “turn” state is only the third most probable cluster, with a population of around 9%. Conversely, in the Aβ1-6_A2V_, while the most populated structure is still an elongated coil (32%), the “turn” configuration is the second most populated structural cluster (31%).

Both Aβ1-6_WT_ and Aβ1-6_A2V_ under physiological conditions are characterized by intramolecular salt bridges such as those between Asp1 and Arg5 or Glu3-Arg5. In the extended coil configuration (Fig. 1E), salt bridges can be dynamically formed and dissociated without requiring a specific rearrangement of the peptide backbone. However, in the turn configuration salt bridges are typically dissociated; the interaction of the apolar Val2 sidechain with the Arg5 sidechain stabilizes such a dissociated state. The additional sterical hindrance to the rearrangement induced by the Val2 sidechain also contributes to the stabilization of the turn configuration of the A2V peptide.

The propensity of the A2V mutant to adopt a Glu3-Arg5 turn configuration characterized by a significant lifetime can be interpreted as the probable source of the heterotypic interaction of the Aβ1-6_A2V_ with full-length Aβ, which results in hindering its assembly.

### Aβ1-6_A2V_ retains the *in vitro* anti-amyloidogenic features of the parental full-length peptide

We previously showed that Aβ1-6_A2V_(D) destabilizes the secondary structure of Aβ1-42_WT_^8^ and is even more effective than the WT peptide [Aβ1-6_WT_(D)] and the A2V-mutated L-isomer [Aβ1-6_A2V_(L)] at preventing the aggregation of full-length Aβ_WT_^8^.

**Figure 2:**
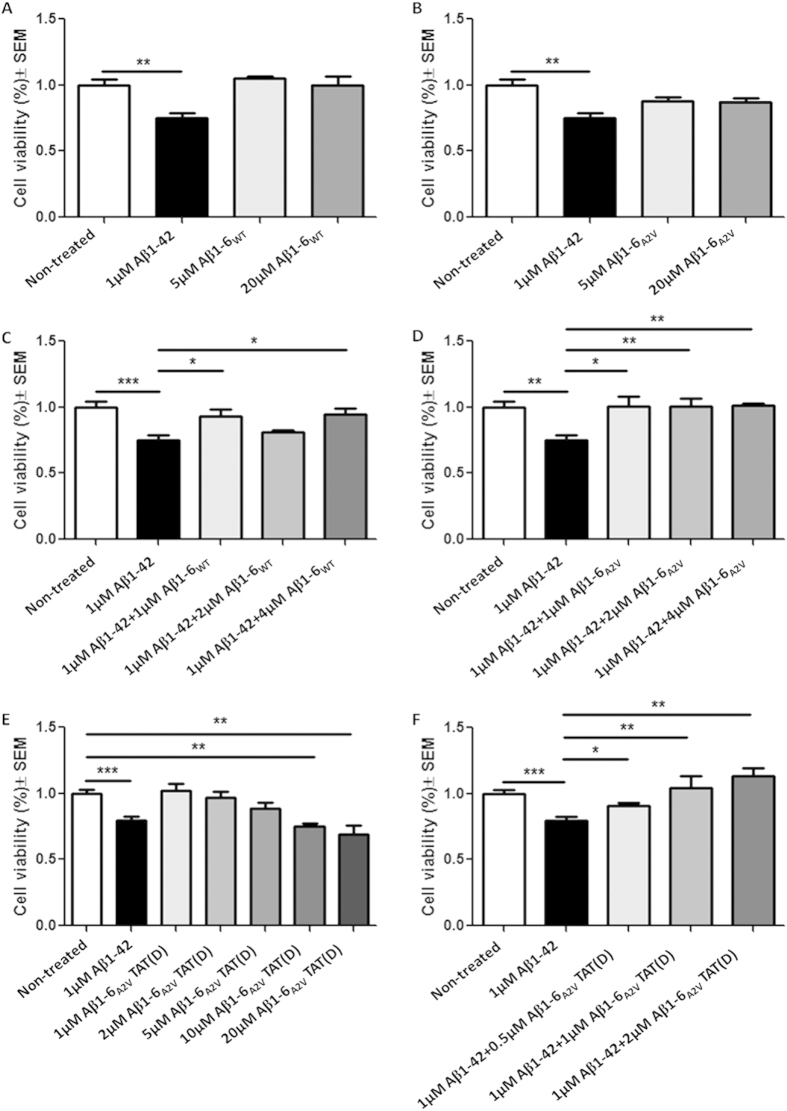
Analysis of the effects of Aβ1-6_WT_(D), Aβ1-6_A2V_(D) and Aβ1-6_A2V_TAT(D) on neurotoxicity in cell models. SH-SY5Y cells were differentiated with 10 μM retinoic acid. After 6 days the proper peptide was added to culture medium and cell viability was assessed after 24 h by MTT test. (**A,B**) Neither Aβ1-6_WT_(D) nor Aβ1-6_A2V_(D) are significantly toxic when added to culture medium of differentiated SH-SY5Y cells. Conversely, Aβ1-42_WT_ reduces cell viability by 35%. * Significance vs non-treated cells. (**C,D**) Both Aβ1-6_WT_(D) and Aβ1-6_A2V_(D) are able to counteract the toxic effect of Aβ1-42_WT_. Aβ1-6_A2V_(D) showed a stronger effect than Aβ1-6_WT_(D). (**E**) Aβ1-6_A2V_TAT(D) is not toxic when added to culture medium at concentrations ranging between 1 and 5 μM, while it reduces cell viability at higher concentrations. * Significance vs non-treated cells. (**F**) Aβ1-6_A2V_TAT(D) showed a dose-dependent effect in reducing Aβ1-42_wt_ toxicity. Comparison of cell viability was performed by Student t-test.

Treatment of SH-SY5Y cells with Aβ1-6_WT_(D) or Aβ1-6_A2V_(D) showed that neither is toxic for living cells even at high concentrations (20 μM) (Fig. 2A,B) and that both peptides are able to reduce the toxicity induced by Aβ1-42_WT_ (Fig. 2C,D). However, Aβ1-6_A2V_(D) showed a stronger effect in counteracting the reduction of cell viability caused by Aβ1-42_WT_ (Fig. 2D), suggesting that the A-to-V substitution actually amplifies the protective effects of the six-mer peptide.

### Aβ1-6_A2V_TAT(D) maintains the *in vitro* anti-amyloidogenic properties of Aβ1-6_A2V_(D)

Aβ1-6_A2V_(D) alone does not efficiently cross either the blood brain barrier (BBB) or cell membranes (data not shown). This is an important feature that would deeply limit its use as an *in vivo* anti-amyloidogenic drug. So, we linked this peptide to the all-D TAT sequence to improve the translocation of Aβ1-6_A2V_(D) across the BBB and cell membranes, minimize the degradation of the peptide and reduce the immune response elicited by the molecule. The resulting compound [Aβ1-6_A2V_TAT(D)] destabilizes the secondary structure of Aβ1-42_WT_. Indeed, CD spectroscopy studies showed that Aβ1-6_A2V_TAT(D) inhibits the acquisition of β-sheet conformation by Aβ1-42_WT_ (data not shown), thus affecting the folding of the full-length peptide.

**Figure 3:**
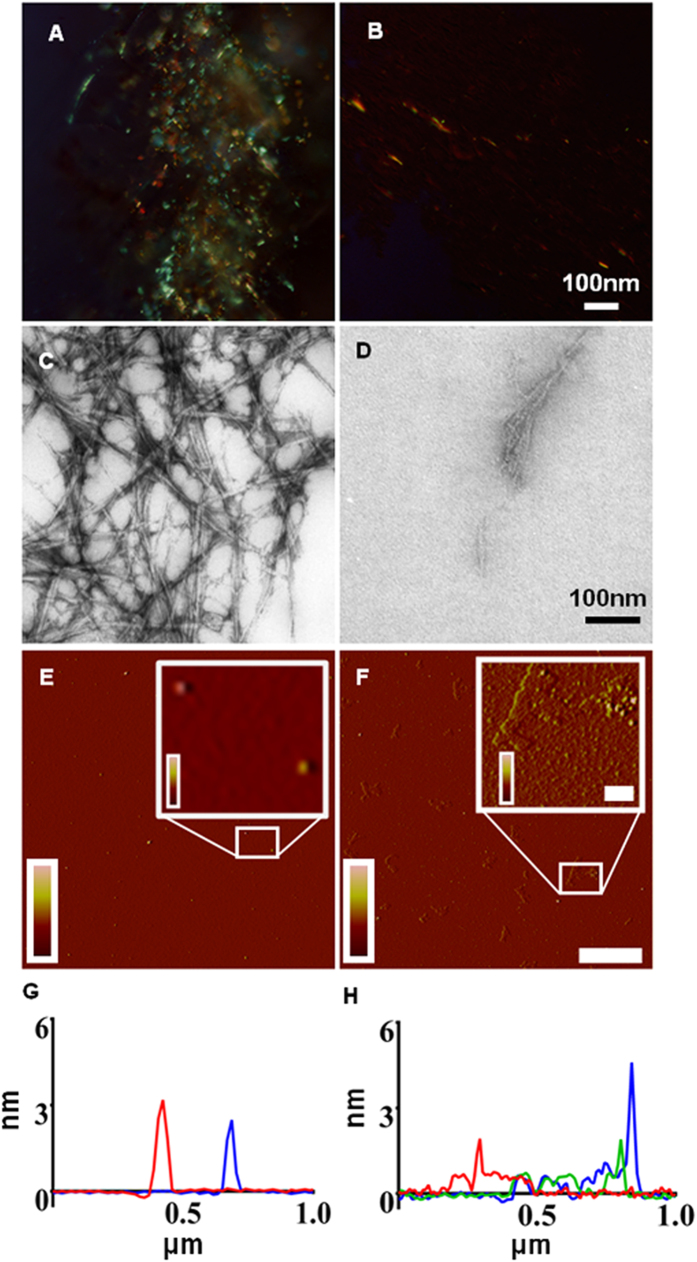
Inhibition of aggregation of Aβ1-42_WT_ by Aβ1-6_A2V_TAT(D). Polarized-light (**A,B**), electron microscopy (**C,D**) and atomic force microscopy (AFM) (**E–H**) studies showing the inhibitory effects of Aβ1-6_A2V_TAT(D) on amyloid formation, fibril production and oligomerization by Aβ1-42_WT_. In polarized-light and EM studies, both peptides were used at 0.125 mM, molar ratio = 1:1 or 1:4 respectively, with 20 days incubation. From 5–20 days, 1:1 co-incubation of the two peptides (**B,D**) displayed a lower amyloid fibril content respect to Aβ1-42_WT_ alone (**A,C**), showing protofibrils, short fibrils and disaggregated granular material. **E,F**: Representative Tapping mode of AFM images as determined by amplitude error data of Aβ1-42_WT_ oligomers. Aβ1-42_WT_ peptide 100 μM in phosphate buffer 50 mM, pH 7.4 was incubated at 4 °C for 24 h alone (**E**) (Z range: -10/ + 10 mV) or in presence of Aβ1-6_A2V_TAT(D) (**F**) (Z range: -10/ + 25 mV). The molar ratio of Aβ1-42_WT_ to Aβ1-6_A2V_TAT(D) was 1:4. Scale bar: 1 μm, inset: 200 nm. (**G,H**): height plot profiles obtained along different lines traced on the topographic AFM images. Overall, these effects were already evident in the 1:1 mixture of the two peptides (data not shown), suggesting that the inhibition of Aβ1-42_WT_ aggregation by Aβ1-6_A2V_TAT(D) is a dose-dependent effect.

We tested the ability of Aβ1-6_A2V_TAT(D) to inhibit the fibrillogenic properties of the full-length Aβ *in vitro* and found that the compound hindered Aβ1-42_WT_ aggregation (Fig. 3). Polarized light and electron microscopy studies on aggregates of Aβ1-42_WT_ formed after 20 days incubation with or without Aβ1-6_A2V_TAT(D) revealed that the mutated peptide hinders the formation of amyloid structures (Fig. 3B) and reduces the amount of fibrils generated by the full-length peptide (Fig. 3D). Moreover, AFM analysis (Fig. 3E,H) showed that Aβ1-6_A2V_TAT(D) actually interferes with the oligomerization process of Aβ1-42_WT_. Indeed, monomeric Aβ1-42_WT_, incubated alone at a final concentration of 100 μM, formed a family of small oligomers of different size within a range of 6-20 nm in diameter (~ 70%) (Fig. 3E,G). Conversely, the co-incubation with Aβ1-6_A2V_TAT(D) resulted in the formation of very small globular structures with a range of 5-8 nm in diameter and height of 200-400 pm (~ 70%), large and thin structures, apparently very rich in water (width: 500–700 nm; height: 200–500 pm). Notably, only rare oligomeric structures were detected (Fig. 3F,H).

These effects were observed by incubating Aβ1-42_WT_ and Aβ1-6_A2V_TAT(D) at a 1:4 molar ratio, but they were also evident at equimolar concentrations of the two peptides.

Moreover, treatment of differentiated SH-SY5Y cells with Aβ1-6_A2V_TAT(D) showed that the peptide is not toxic when administered at concentrations ranging between 1 and 5 μM (Fig. 2E). When co-incubated with Aβ1-42_WT_, Aβ1-6_A2V_TAT(D) displayed a significant dose-dependent reduction of the toxicity induced by full-length Aβ (Fig. 2F).

All these findings indicated that the designed Aβ1-6_A2V_TAT(D) peptide is particularly efficient at inhibiting Aβ polymerization and toxicity *in vitro*, and identified it as our lead compound for the subsequent *in vivo* studies.

### Aβ1-6_A2V_TAT(D) is able to cross the BBB after its intraperitoneal administration

**Figure 4:**
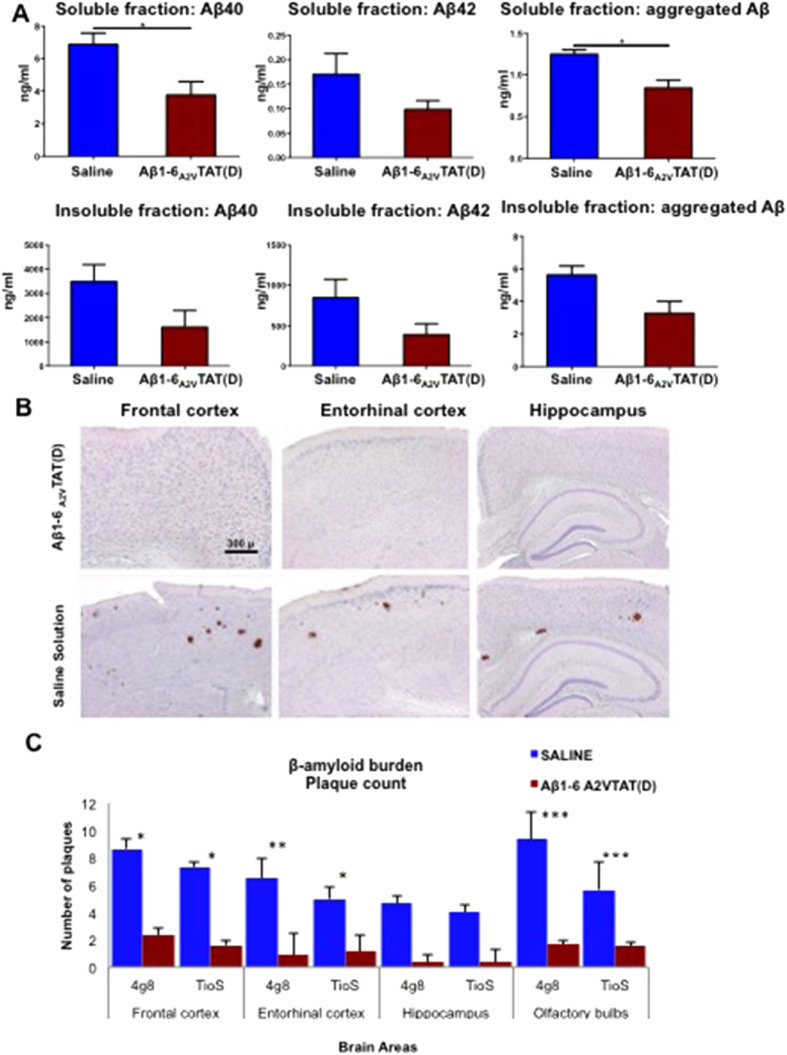
*In vivo* anti-amyloidogenic effects of treatment with Aβ1-6_A2V_TAT(D) – pilot study in huAPP^Swe^/moAPP^0/0^ mice. (**A**): Effects of Aβ1-6_A2V_TAT(D) treatment on brain levels of full-length Aβ and Aβ aggregates in huAPP^Swe^/moAPP^0/0^ mice. Mice treated i.p. with Aβ1-6_A2V_TAT(D) daily for 21 days showed a reduction of Aβ40 levels in the soluble fraction, together with a decrease in aggregated Aβ. Aβ42 in the soluble fraction and Aβ40, Aβ42 and aggregated Aβ in the insoluble fraction were not significantly lower, even though we observed a trend of reduction of these species in mice treated with Aβ1-6_A2V_TAT(D) as compared to animals treated with saline solution. Student t-test was used to compare results obtained by ELISA tests. (**B,C**) Prevention of *in vivo* amyloid formation in huAPPSwe/moAPP0/0 mice by short-term treatment with Aβ1-6_A2V_TAT(D). (**B**) Amyloid deposits in mice treated with Aβ1-6_A2V_TAT(D) vs mice treated with saline solution (control group). Immunohistochemistry with 4G8 antibody, original magnification 4×. (**C**) Quantification of amyloid burden by ‘plaque count’ in the two experimental groups: saline treated animals (blue columns) and Aβ1-6_A2V_TAT(D)-treated mice (red columns). Evidence of reduction of amyloid burden in the group treated with Aβ1-6_A2V_TAT(D). Significance of the results was calculated by Mann-Whitney U test. Statistical differences were considered significant if p < 0.05.

The stability of Aβ1-6_A2V_TAT(D) and Aβ1-6_A2V_TAT(L) peptides to serum proteases was determined using MALDI-TOF. The D-peptide remained stable for 48 h, while the L-peptide was rapidly degraded in about 1 h (Supplementary Fig. 3 S). To check the efficacy of TAT peptide in delivering Aβ1-6_A2V_ to the brain, we measured the levels of Aβ1-6_A2V_TAT(D) in brain tissue of mice treated with the compound i.p. once a week at the dose of 10 mg/kg for five months, 1 or 24 h after the last treatment. The analysis showed that Aβ1-6_A2V_TAT(D) brain levels were 145.03 ± 38.26 pg/mg tissue (mean ± SEM) and 62.02 ± 28.68 after 1 or 24 h, respectively (Supplementary Table 3S and Fig. 4 S).

### Aβ1-6_A2V_TAT(D) shows *in vivo* anti-amyloidogenic effects in the huAPP^Swe^/moAPP^0/0^ mouse model of AD

A pilot preclinical study with Aβ1-6_A2V_TAT(D) was performed on a limited number (n = 3) of transgenic mice - APP23 mice expressing the Swedish double mutation in the human APP gene and knock-out for endogenous APP (huAPP^Swe^/moAPP^0/0^), chosen to avoid the interference of murine APP in the anti-amyloidogenic properties of Aβ1-6_A2V_TAT(D). These mice usually begin to develop amyloid deposits at 10 months. The animals were treated i.p. at 13 months with 0.24 mg/kg of Aβ1-6_A2V_TAT(D) once a day for 3 weeks. The treatment resulted in a reduction of Aβ production and aggregation (Fig. 4A) and in a decrease of intracerebral amyloid deposits (Fig. 4B,C).

### Aβ1-6_A2V_TAT(D) reduces Aβ levels and amyloid in APPswe/PS1dE9 mice in a short treatment schedule

Ultimately, we decided to treat APPswe/PS1dE9 mice (n = 20 x group) i.p. with Aβ1-6_A2V_TAT(D). The treatment started at the age of 4 months, when the deposition of amyloid in the brain of these animals usually begins, becoming consistent by 6 months. The schedule of treatment was established based on the results of a pilot study with the same mouse model, showing that the best anti-amyloidogenic effects are obtained by treating animals i.p. once a week with 10 mg/kg peptide (data not shown).

**Figure 5:**
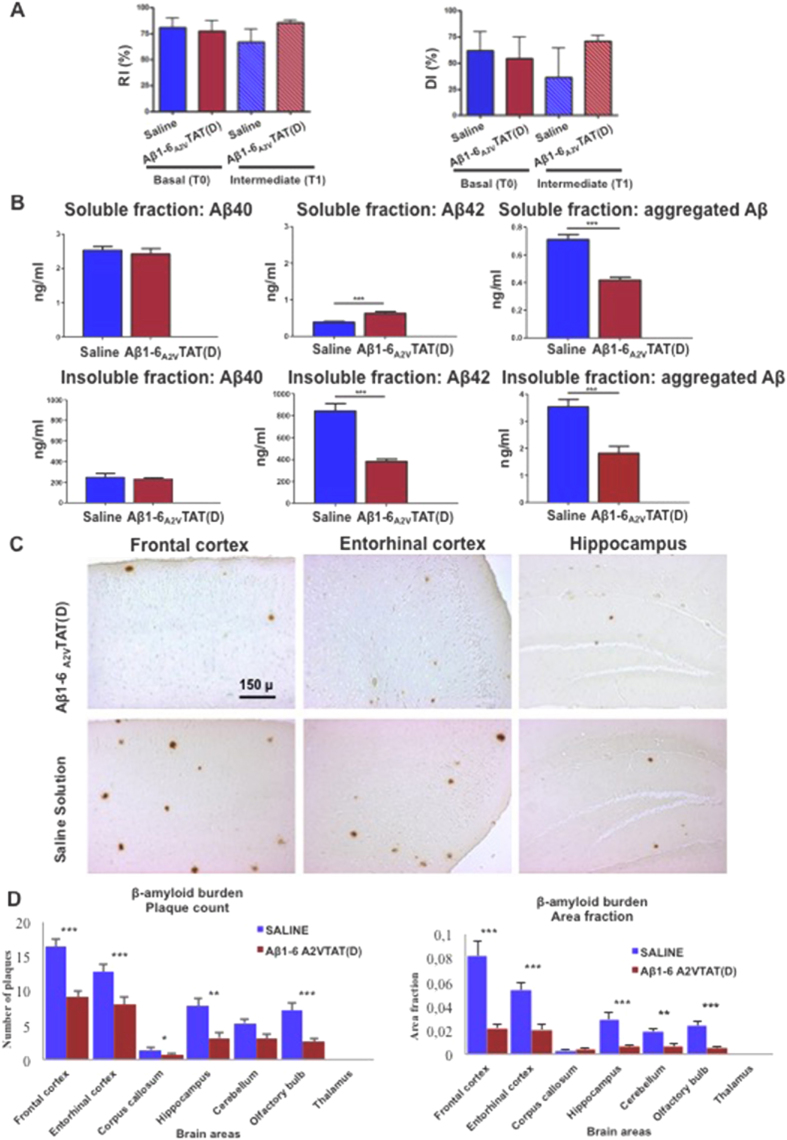
*In vivo* anti-amyloidogenic effects of a short schedule treatment with Aβ1-6_A2V_TAT(D). (**A**) Novel Object Recognition Test (NOR). Recognition/Discrimination Index at the end of treatment with Aβ1-6_A2V_TAT(D). Animals were treated with Aβ1-6_A2V_TAT(D) once a week for 2.5 months. Comparison with control group (treated with saline solution). The significance of the results was calculated via a Mann-Whitney U test. (**B**) Biochemical study on mice treated with Aβ1-6_A2V_TAT(D) once a week for 2.5 months. Aβ levels in the brains of mice treated with Aβ1-6_A2V_TAT(D) every 7 days for 2.5 months showed an increase of Aβ42 in the soluble fraction and a concomitant decrease in the insoluble fraction. Interestingly, aggregated Aβ was reduced in both the compartments. Aβ40 was not changed compared to controls. Statistical analysis was performed via a Student’s t-test and differences were considered significant if p < 0.05. (**C,D**) Neuropathological study on mice treated with Aβ1-6_A2V_TAT(D) once a week for 2.5 months. Amyloid deposits in mice treated with Aβ1-6_A2V_TAT(D) vs mice treated with saline solution (control group). Immunohistochemistry with 4G8 antibody, original magnification 10× (**C**). Quantification of amyloid burden by ‘plaque count’ and ‘area fraction’ in the two experimental groups: saline treated animals (blue columns) and Aβ1-6_A2V_TAT(D)-treated mice (red columns) (**D**). Evidence of reduction of amyloid burden in the group treated with Aβ1-6_A2V_TAT(D). The significance of the results was calculated via a Mann-Whitney U test. Statistical differences were considered significant if p < 0.05.

The short-term treatment (2.5 months) of APPswe/PS1dE9 mice with Aβ1-6_A2V_TAT(D) resulted in the exciting reduction of Aβ production and aggregation (Fig. 5B), and prevention of amyloid deposition in the brain (Fig. 5C,D) of the group treated once a week with Aβ1-6_A2V_TAT(D). This group showed an increase of Aβ1-42 in the soluble fraction and a concomitant decrease in the insoluble fraction, suggesting that the treatment induces a transfer of Aβ1-42 from the insoluble to soluble compartment of brain tissue, which may reflect a disaggregation of amyloid deposits. This view is corroborated by the fact that aggregated Aβ was reduced in both compartments. Moreover, the decrease of aggregated Aβ in the soluble fraction is associated with an increase of monomeric Aβ1-42 levels, suggesting that A2V-based therapy changes the dynamic equilibrium between Aβ aggregates and Aβ monomers, resulting in a reduction of the most toxic Aβ species (i.e., soluble Aβ assemblies).

The reduction of amyloid deposition involved all brain areas, including the hippocampus, and was more prominent in the frontal and entorhinal cortices and olfactory bulbs (Fig. 5D).

These findings were paralleled by a slight effect on cognitive function (Fig. 5A), as suggested by performances of mice treated with Aβ1-6_A2V_TAT(D) in the Novel Object Recognition test (NOR). The results of the study showed a trend towards cognitive impairment in the control group and an opposite tendency of mice treated with Aβ1-6_A2V_TAT(D), whose cognitive performances were no worse than at the beginning of the treatment with Aβ1-6_A2V_TAT(D). In fact, the NOR test scores showed a small, albeit not statistically significant, improvement.

**Figure 6:**
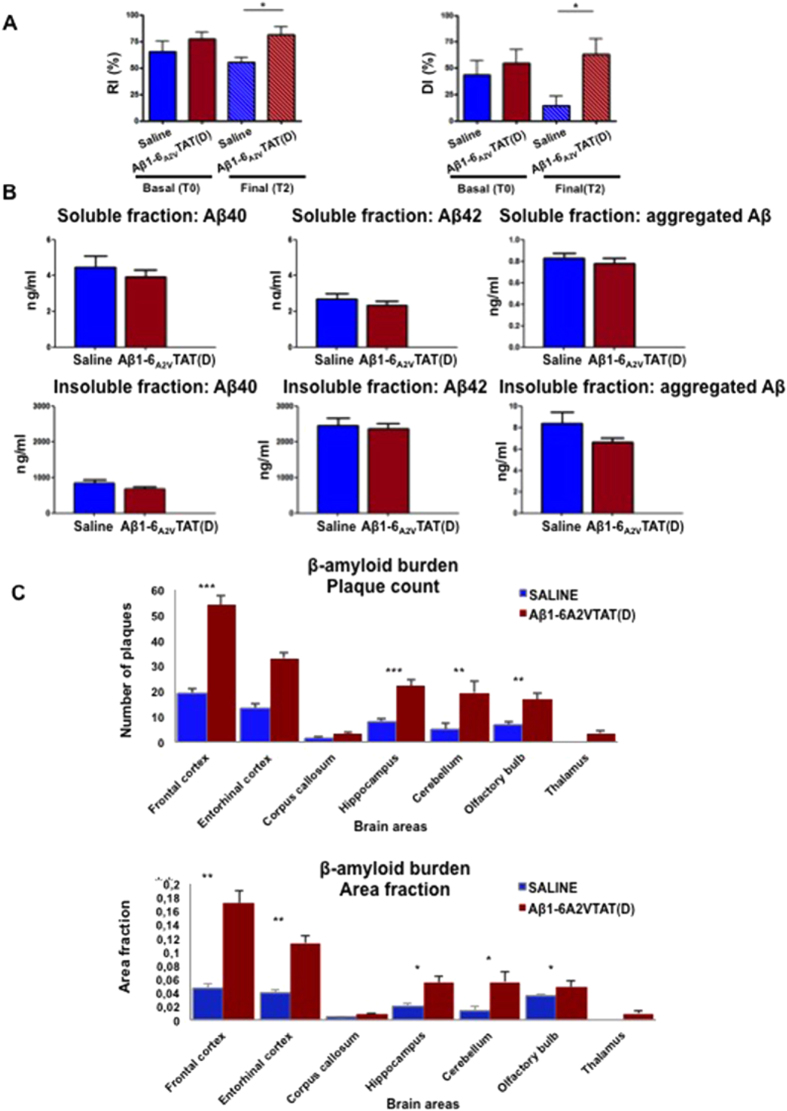
*In vivo* anti-amyloidogenic effects of prolonged treatment with Aβ1-6_A2V_TAT(D). (**A**): Novel Object Recognition Test (NOR). Recognition/Discrimination Index at the end of treatment with Aβ1-6_A2V_TAT(D). Animals were treated with Aβ1-6_A2V_TAT(D) once a week for 5 months. Comparison with control group (treated with saline solution) . The significance of the results was calculated using a Mann-Whitney U test. (**B**) Biochemical study on mice treated with Aβ1-6_A2V_TAT(D) once a week for 5 months. Aβ levels in the brains of mice treated with Aβ1-6_A2V_TAT(D) every 7 days for 5 months showed no significant difference compared to mice treated with saline solution. However, Aβ40, Aβ42 and aggregated Aβ levels were slightly lower in both soluble and insoluble fraction of mice treated with Aβ1-6_A2V_TAT(D). Statistical analysis was performed via a Student’s t-test and differences were considered significant if p < 0.05. (**C**) Neuropathological study on mice treated with Aβ1-6_A2V_TAT(D) once a week for 5 months Quantification of amyloid burden by ‘plaque count’ and ‘area fraction’ in the two experimental groups: saline-treated animals (blue columns) and Aβ1-6_A2V_TAT(D)-treated mice (red columns). Amyloid burden was unexpectedly increased in the group treated with Aβ1-6_A2V_TAT(D) in comparison with mice treated with saline solution (control group). The significance of the results was calculated using a Mann-Whitney U test. Statistical differences were considered significant if p < 0.05.

However, the final outcome of treatment (after 5 months) was an unexpected substantial increase in amyloid burden (Fig. 6C) and attenuation of the effects on Aβ production (Fig. 6B), while the results of the behavioral assessment by NOR suggested preserved cognitive function in the group treated with Aβ1-6_A2V_TAT(D) compared with the saline-treated mice, as indicated by significant differences in the scores for recognition and discrimination indexes obtained for the two experimental group of mice at the end of treatment (Fig. 6A).

### Aβ1-6_A2V_TAT(D) induces a shift in APP processing toward the amyloidogenic pathway in a chronic treatment

The unexpected outcome of the prolonged treatment with Aβ1-6_A2V_TAT(D) spurred us to search for the causes underlying the removal of the anti-amyloidogenic effects previously observed after 2.5 months of treatment with the test compound.

**Figure 7:**
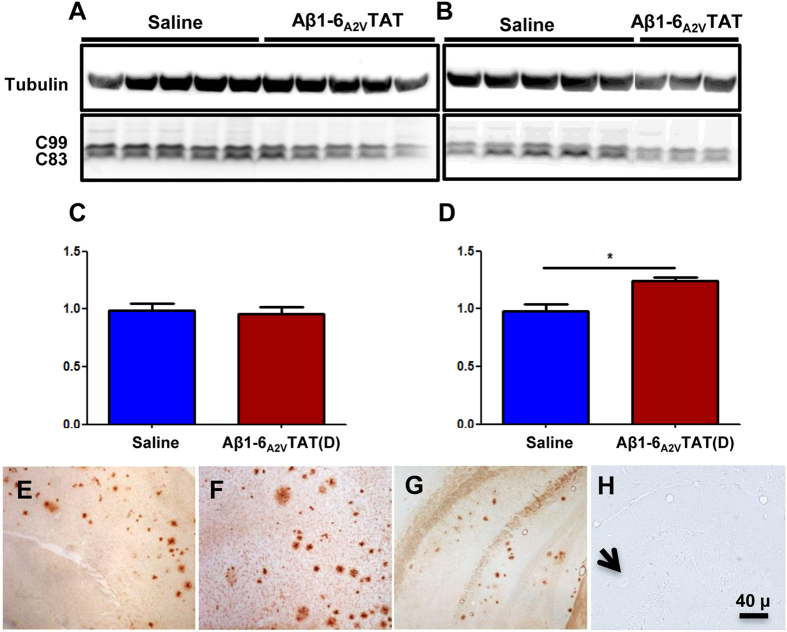
Effects of TAT(D) on APP processing (A–D) and binding of peptides to amyloid deposits (E–H). Densitometric quantification of APP C-terminal fragments revealed an increase in the C99:C83 ratio in mice treated with Aβ1-6_A2V_TAT(D) every 7 days for 5 months (**B,D**), while no differences in C99:C83 ratio were observed at the beginning of the treatment (**A,C**). Statistical analysis was performed using a Student’s t-test and differences were considered significant if p < 0.05. Immunohistochemistry with biotinylated peptides, original magnification 20×. Amyloid plaque immunohistochemistry in brain sections of CRND8 transgenic mice. **E** and **F** show transgenic mouse brain stained with Biot-Aβ1-6_WT_TAT(D) and Biot-Aβ1-6_A2V_TAT(D) respectively. (**G**): transgenic mouse brain stained with Biot-TAT(D). (**H**): transgenic mouse brain stained with Biot-Aβ1-6_A2V_(D). Arrow indicates a plaque that is not stained by Biot-Aβ1-6_A2V_. No immunoreactivity was detected in control non-transgenic mice slides co-incubated with Biot-D peptides (data not shown).

To this end, we investigated the effects of Aβ1-6_A2V_TAT(D) on APP processing and found that treatment with Aβ1-6_A2V_TAT(D) resulted in a shift of APP processing towards the amyloidogenic pathway, leading to an increased C99:C83 ratio in the brain of APPswe/PS1dE9 mice (Fig. 7A–D), which presumably paves the way for overproduction of Aβ. Interestingly, the effects on APP processing were detected only after prolonged treatment with Aβ1-6_A2V_TAT(D) and were not observed after short-term treatment schedules, i.e., after 3 weeks in the huAPP^Swe^/moAPP^0/0^ or after 2.5 months in the APPswe/PS1dE9 mice.

### TAT peptide alone binds amyloid vigorously

Concomitant studies performed in our labs showed that Aβ1-6_A2V_TAT(D) has a special propensity to target β-amyloid deposits (Fig. 7E–H) and that this is a specific attribute of the TAT(D) sequence.

To address this point, we prepared Aβ1-6_A2V_TAT(D), Aβ1-6_WT_TAT(D), Aβ1-6_A2V_(D) and TAT(D) peptides containing a biotinylated residue at the C-terminus (Biot-peptide) to visualize their binding to amyloid plaques. Both Biot-Aβ1-6_WT_TAT(D) and Biot-Aβ1-6_A2V_TAT(D) were unable to produce any staining in brain slices of control animals (non-transgenic mice) devoid of amyloid plaques (data not shown), but they were able to bind and therefore stain the plaques in transgenic mouse brains (Fig. 7E,F respectively). Interestingly, the biotinylated form of TAT(D) [(Biot-TAT(D)] strongly stained amyloid deposits (Fig. 7G), but no immunostaining of amyloid was detected in slices incubated with Biot-Aβ1-6_A2V_(D) (Fig. 7H).

These data suggest that the anti-amyloidogenic effects of Aβ1-6_A2V_(D) are overcome in the chronic treatment by the TAT intrinsic attitude to promote amyloidogenic APP processing, bind to amyloid and boost its seeding activity, leading to an increase of amyloid burden *in vivo*.

### Aβ1-6_A2V_TAT(D) induces an immune response after peripheral administration in mice

**Figure 8:**
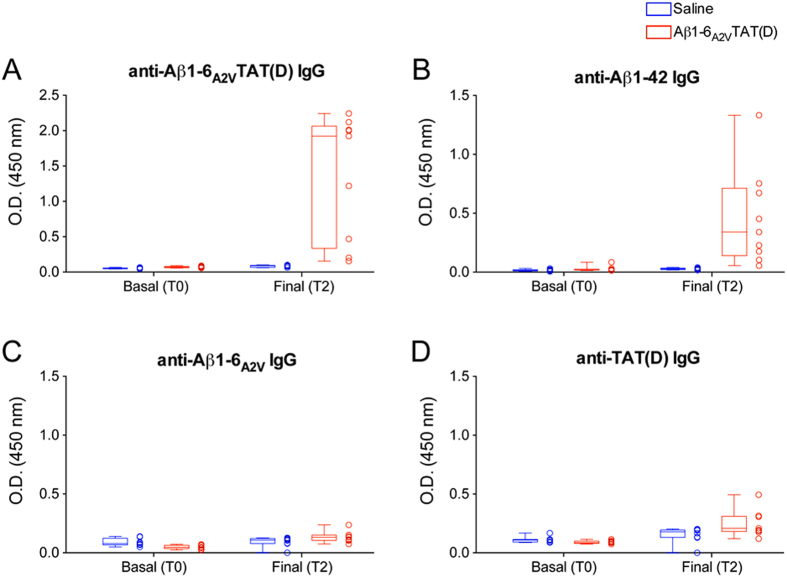
Serum levels of IgG against Aβ peptides after chronic treatment with Aβ1-6_A2V_TAT(D). To verify whether Aβ1-6_A2V_TAT(D) administration once a week for 5 months was associated with an immune response, we collected blood from mice before (T0) and at the end of the treatment (T2). Sera from saline (n = 8) and Aβ1-6_A2V_TAT(D) (n = 9) treated mice were tested induplicate using ELISA for the presence of IgG against Aβ1-6_A2V_TAT(D) (**A**), the full-length Aβ1-42 (**B**), Aβ1-6_A2V_ (**C**) and TAT(D) (**D**). Data are displayed both as the median (range) of each group and as individual values and represent optical density (O.D.) at 450 nm.

Finally, we tested the Aβ1-6_A2V_TAT(D) immunogenic properties and found that the chronic treatment with this compound results in the production of not negligible levels of serum IgG against Aβ1-6_A2V_TAT(D) as well as against full-length Aβ (Fig. 8A,B). Interestingly, the treatment with Aβ1-6_A2V_TAT(D) did not induce production of IgG against Aβ1-6_A2V_ peptide (Fig. 8C), while 3 out of 9 mice displayed slightly increased levels of IgG against TAT(D) (Fig. 8D). These data suggest that both Aβ1-6_A2V_ and TAT(D) are not *per se* strongly immunogenic, while the combination of the two peptides might confer strong immunogenicity to Aβ1-6_A2V_TAT(D) compound that, however, did not result in any evident side effect on brain, as revealed by neuropathologic studies (data not shown).

## Discussion

During the last few decades, huge efforts have been made to develop disease-modifying therapies for Alzheimer, but the results of these attempts have been frustrating. The anticipated increase of AD patients in the next few decades makes the development of efficient treatments an urgent issue^16^. In order to prevent the disease and radically change its irreversible course, a long series of experimental strategies against the main molecular actors of the disease (Aβ and tau)^17^ or novel therapeutic targets^18^ have been designed based on purely theoretical grounds^19^ as well as on evidence mainly deriving from preclinical observations in AD animal models^20^. However, few strategies proved suitable for application in human clinical trials, and none proved to be really effective^21^.Our approach differs from previous strategies - mainly those involving modified Aβ peptides that have been found to inhibit amyloidogenesis^19^,^22^ - since it is based on a natural genetic variant of amyloid-β (Aβ_A2V_) that occurs in humans and prevents the development of the disease when present in the heterozygous state^7^.

In this context, we carried out *in vitro* and *in vivo* studies that revealed the extraordinary ability of Aβ_A2V_ to interact with Aβ_WT_, interfering with its aggregation^8^. These findings were a proof of concept of the validity of therapeutic strategies based on the use of Aβ_A2V_ variant, and prompted us to develop a new disease-modifying treatment for AD by designing a six-mer mutated D-isomer peptide [Aβ1-6_A2V_(D)] linked to the short amino acid sequence derived from the HIV TAT peptide, widely used for brain delivery, to make the translocation of Aβ1-6_A2V_(D) across the BBB feasible.

The use of TAT as a carrier for brain delivery of drugs has been employed in several experimental approaches for the treatment of AD-like pathology in mouse models^12^,^13^. Recently, intraperitoneal administration of a TAT-BDNF peptide complex for 1 month was shown to improve the cognitive functions in AD rodent models^23^.

A previous study showed that, following its peripheral injection, a fluorescein-labelled version of TAT is able to cross the BBB, bind amyloid plaques and activate microglia in the cerebral cortex of APPswe/PS1DE9 transgenic mice^24^. TAT was then conjugated with a peptide inhibitor (RI-OR2, Ac-rGffvlkGr-NH2) consisting of a retro-inverted version of Aβ16–20 sequence^25^ that was found to block the formation of Aβ aggregates *in vitro* and to inhibit the toxicity of Aβ on cultured cells^25^. Daily i.p. injection of RI-OR2-TAT for 21 days into 10-month-old APPswe/PS1DE9 mice resulted in a reduction in Aβ oligomer levels and amyloid-β burden in cerebral cortex^24^.

We followed a similar strategy and initially demonstrated that Aβ1-6_A2V_(D), with or without the TAT sequence, retains *in vitro* the anti-amyloidogenic properties of the parental full-length mutated Aβ, since it is effective at hindering *in vitro* the production of oligomers and fibrils, the formation of amyloid and the toxicity induced by Aβ1-42_WT_ peptide on SYSH-5Y cells.

Based on these results, we then decided to test *in vivo* the anti-amyloidogenic ability of Aβ1-6_A2V_TAT(D). The compound proved stable in serum after i.p. administration in mice, able to cross the BBB and associated with an immune response that was not found to cause any brain damage.

Short-term treatment with Aβ1-6_A2V_TAT(D) in the APPswe/PS1DE9 mouse model prevented cognitive deterioration, Aβ aggregation and amyloid deposition in brain. Unexpectedly, a longer treatment schedule, while retaining the results for cognitive impairment, attenuated the effects on Aβ production and increased amyloid burden, most likely due to the intrinsic amyloidogenic properties of TAT.

Indeed, we found that TAT(D), unlike Aβ1-6_A2V_(D), has a strong ability to bind amyloid deposits. This avidity for amyloid could boost the intrinsic seeding activity of amyloid plaques via a continuous and self-sustained recruitment of Aβ aggregates, leading to an exacerbation of the amyloidogenesis.

A similar effect of TAT was described in a study^26^ reporting that HIV TAT promotes AD-like pathology in an AD mouse model co-expressing human APP bearing the Swedish mutation and TAT peptide (PSAPP/TAT mice). These mice indeed showed more Aβ deposition, neurodegeneration, neuronal apoptotic signalling, and phospho-tau production than PSAPP mice.

Moreover, TAT was found to increase Aβ levels by inhibiting neprilysin^27^ or enhancing β-secretase cleavage of APP, resulting in increased levels of the C99 APP fragment and 5.5-fold higher levels of Aβ42^28^. The same study reported that stereotaxic injection of a lentiviral TAT expression construct into the hippocampus of APP/presenilin-1 (PS1) transgenic mice resulted in increased TAT-mediated production of Aβ *in vivo* as well as an increase in the number and size of Aβ plaques. This is consistent with our findings, indicating a shift in APP processing towards the amyloidogenic processing *in vivo* at the end of the 5-month treatment with Aβ1-6_A2V_TAT(D) that was not observed in shorter treatment schedules with the same compound.

Therefore, these data suggest that the final outcome of our *in vivo* studies with Aβ1-6_A2V_TAT(D) is the result of side effects of the TAT carrier, whose amyloidogenic intrinsic activity neutralized the anti-amyloidogenic properties of the Aβ_A2V_ variant. Nevertheless, we believe that the approach based on the use of Aβ_A2V_ variant can be successfully used in treating AD, because of its potential ability to tackle the main pathogenic events involved in the disease, as suggested by the natural protection against the disease which occurs in human heterozygous A673V carriers.

Interestingly, recent studies produced evidence in favour of a natural protection against AD in human carriers of the A2T Aβ mutation^29^, another human Aβ variant characterized by an alanine-to-threonine substitution at the same APP codon of the A2V-Aβ mutation (APP-A673T or Aβ_A2T_ variant). This mutation segregates almost exclusively with individuals who never showed any sign of dementia, suggesting a possible protective role against AD in the Icelandic and Finnish populations^30^. Additional studies tried to clarify the molecular basis of the A2T-induced protection for AD, suggesting a likely composite mechanism including effects on APP processing (with consequent decrease of Aβ production), and on Aβ structure, aggregation and neurotoxicity^31^,^32^,^33^.

More recent papers reported that the A673T variant is extremely rare in other cohorts from the US and Asia and does not play a substantial role in risk for AD outside of Icelandic and Scandinavian geographic areas^34^,^35^. However, the discovery of protective genetic variants like Aβ_A2V_ and Aβ_A2T_, although rare, should prompt a novel vision of genetic studies, which until now has been limited to the identification of pathogenic variants, expanding the genetic research into the detection of “protective” DNA variations as a useful foundation for the design of efficient disease-modifying treatments in medicine.

Finally, we would emphasize that, regardless its optimal BBB delivery abilities and cell penetrating activity^36^, some intrinsic properties of TAT sequence can promote amyloidogenesis in long treatment schedules and should be carefully taken into account whenever this peptide is employed in the design of therapeutic strategies for neurological diseases^37^,^38^,^39^, particularly AD^40^.

## Materials and Methods

### In silico studies

The impact of the A2V mutation on the structural features of the Aβ1-6 peptide was investigated using classical molecular dynamics (MD) simulations with an all-atom approach using the AMBER ff99SB-ILDN force field^41^. The solvent environment was explicitly simulated using the Tip3p water model and applying three-dimensional periodic boundary conditions. A 1.0 nm cutoff for the direct calculation of non-bonded interactions was applied, and long-range electrostatics were treated with the particle-mesh Ewald approach. The systems were prepared by dispersing the Aβ1-6_WT_ and Aβ1-6_A2V_ peptides in a cubic box of side 5.0 nm, filled with water molecules, and were initially relaxed through energy minimization in order to avoid nonphysical contact between the solute and solvent molecules, then equilibrated with a 5 ns MD simulation at constant ambient pressure and temperature (300 K). The production runs were then extended for 1.5 μs each. Covalent bonds involving hydrogen were constrained by applying the LINCS algorithm, allowing a simulation timestep of 2 fs^42^. Temperature was controlled by applying the Bussi-Donadio-Parrinello thermostat, while pressure was controlled with an isotropic Parrinello-Rahman barostat^43^. All simulations were carried out using GROMACS 4.6.5^42^.

### *In vitro* studies

#### Peptide synthesis

All synthetic peptides were prepared as previously described^7^ via solid-phase Fmoc chemistry on an Applied Biosystems 433 A peptide synthesizer, characterized by matrix-assisted laser desorption/ionization mass spectrometry (MALDI). Their purity was always above 95% (See Supplementary Information for details).

#### Polarized light, Electron Microscopy (EM)

Aβ1-42_WT_, Aβ1-6_A2V_TAT(D) were dissolved in 10 mM NaOH and then diluted in an equal volume of 100 mM Tris-HCl, pH 7.0, to final concentrations (0.250 mM). In studies with peptide mixtures [Aβ1-42_WT_ : Aβ1-6_A2V_TAT(D)], peptide solutions were prepared as described above at a final concentration of 0.250 mM at either 1:1 or 1:4 molar ratios. The samples were incubated at 37 °C, and the tinctorial and ultrastructural properties of the peptide assemblies were determined at various intervals, ranging from 1 h to 20 days, as previously described^7^. The experiments were repeated five times.

#### Atomic Force Microscopy (AFM)

Aβ1-42_WT_ oligomers were formed following incubation of Aβ1-42_WT_ at a final concentration of 100 μM in phosphate buffer 50 mM, pH 7.4 at 4 °C for 24 h, alone or in presence of Aβ1-6_A2V_TAT(D), at 1:4 molar ratio. Sample solutions were diluted to a final concentration of 10 μM of Aβ1-42_WT_ with phosphate buffer and 50 μl of each solution was immediately added onto freshly cleaved mica at room temperature for 2 minutes. Samples were washed and dried under gentle nitrogen flow. AFM analyses were carried out on a Multimode AFM with a Nanoscope V system (Veeco/Digital Instruments). AFM images were analysed using the Scanning Probe Image Processor data analysis package. All the topographic patterns and SPIP characterization were verified via additional measurements on a minimum of five different, well separated areas.

#### Binding of Biot-Aβ1-6_WT/A2V_TAT(D) to amyloid

It was tested on brain sections of transgenic and control mice of CRND8 strain^44^. To unequivocally identify the fragment(s) of the peptide responsible for binding to plaques we used three separate segments: Biot-Aβ1-6_WT_(D), Biot-Aβ1-6_A2V_(D) and Biot-TAT(D). Seven-micrometre thick brain sections fixed in Carnoy and embedded in paraffin were used for staining. The immunoreaction was revealed by incubation with the avidin-biotin complex (Vectastatin ABC Kit, Vector Laboratories) and diaminobenzidine (DAB) as the substrate for horseradish peroxidase.

#### Cell models

Toxicity studies with Aβ1-6_WT_(D), Aβ1-6_A2V_(D) or Aβ1-6_A2V_TAT(D) and the short peptides’ ability to inhibit the toxicity induced by Aβ1-42_WT_ in SH-SY5Y cell cultures were analyzed following previously described protocols^7^. Each experiment was performed in triplicate; the data provided were obtained as the mean of three independent assays.

### *In vivo* studies

#### Animals

Animal care and experimental procedures were conducted in accordance with European Union (2010/63/EU) and Italian (D. Lgs. 26/2014) legislations and followed the applicable rules and guidelines of the Animal care surveillance Committee of the INCB. All the experiments involving animals were approved by the Animal care surveillance Committee of the INCB and by the Italian Ministry of Health. All animals were sacrificed by cervical dislocation under deep anesthesia (telazol 20 mg/kg and medetomidine 1,5 mg/kg i.p.).

#### *In vivo* assessment of translocation across BBB

APP23 mice (Harlan, Correzzana, Italy) were treated by intraperitoneal injection with Aβ1-6_A2V_TAT(D) (10 mg/kg) or vehicle (saline solution) and sacrificed 1 h or 24 h after the last treatment. Aβ1-6_A2V_TAT(D) was quantified in mouse brain using HPLC-MS/MS^45^. The stability of Aβ1-6_A2V_TAT(D) to protease digestion was also investigated by MALDI TOF MS (See Supplementary Information for details).

#### *In vivo* anti-amyloidogenic effects of Aβ1-6_A2V_TAT(D) in the huAPP^Swe^/moAPP^0/0^ mouse model

Transgenic APP23 mice expressing the Swedish double mutation in the human APP gene^46^ and knock-out for endogenous APP (huAPP^Swe^/moAPP^0/0^) were generated in our lab by crossing APP23 mice with APP^0/0^ animals. Mice (n = 6) were treated intraperitoneally (i.p.) every day for 21 days with Aβ1-6_A2V_TAT(D) (10 mg/kg) or saline solution and culled 24 h after the last treatment.

#### *In vivo* anti-amyloidogenic effects of Aβ1-6_A2V_TAT(D) in the APPswe/PS1dE9 mouse model

Heterozygous four-month-old female APPswe/PS1dE9 transgenic mice were used in this study. These mice express human APP carrying the Swedish mutation together with human presenilin 1 (PS1) carrying the pathogenic dE9 mutation^47^. Animals were divided in 2 experimental groups (n = 10 each), treated i.p. once a week with saline solution or Aβ1-6_A2V_TAT(D) and culled 2.5 (intermediate time) and 5 months (final time) after treatment. In both mouse transgenic lines, the brain was removed and dissected into 2 hemibrains by midsagittal dissection. One half was immediately stored at 80 °C for biochemical assays, the other immediately immersed in formalin (10%) overnight for immunohistochemical studies.

#### Behavioral tests

The Novel Object Recognition test (NOR) was used to examine the cognitive performance of animal models involved in preclinical studies, following well consolidated protocols^48^. The results of the NOR were expressed by using:

- Discrimination index (time exploring novel object - time exploring familiar object)/(time exploring novel object + time exploring familiar object).

- Recognition index (RI): time exploring novel object/(time exploring novel object + time exploring familiar object) %.

#### Biochemical studies

The left hemisphere of each brain was homogenized in 7 volumes of 10 mM Tris-HCl, pH 7.4, added with cOmplete Mini Protease Inhibitors cocktail (Roche), sonicated for 1 min using an ultrasonic homogenizer (SONOPULS) and centrifuged at 100,000 xg for 1 h at 4 °C. The supernatant was saved as the soluble fraction; the pellet was extracted in 70% formic acid and neutralized with 20 volumes of 1 M Tris (insoluble fraction).

Aβ40, Aβ42 and aggregated Aβ were measured in both soluble and insoluble fractions by ELISA (Invitrogen). Each experiment was performed in triplicate. The analysis of APP processing followed the protocol described above. After centrifugation, the supernatant was collected as the SDS-soluble fraction, which was analyzed by western blot with the A8717 antibody (Sigma). Signal intensity of the bands was measured by Quantity One (BioRad).

#### Neuropathological studies

Coronal slices of the right hemibrain were embedded in paraffin and cut (7μm); sections were de-waxed in xylene and hydrated through serial alcohols to water. After formic acid (80%) pre-treatment, sections were incubated overnight with anti-Aβ antibody (4G8, 1:4000; Covance). The primary antibody signal was detected with a biotinylated secondary antibody followed by horseradish streptavidin peroxidase and visualized with DAB. Amyloid deposition was quantified in mouse brain using Aβ immunostaining (4G8) and the staining intensity was evaluated semiquantitatively using a scale range from 0 to 5 by light microscopy. The assessment was conducted in two adjacent sections of the same brain area^49^. A parallel quantification of the Aβ plaque load was performed using image analysis software (NIS-elements-Nikon)^50^,^51^,^52^.

#### Measurement of serum antibodies against Aβ peptides

Sera were collected from mice before (T0) and at the end of the treatment (5 months), and tested using ELISA for the presence of IgG against Aβ1-6_A2V_TAT(D), the full-length Aβ1-42, Aβ1-6_A2V_(D) and TAT(D) following previously described protocols for antigen-specific mouse IgG detection^53^, except blocking buffer that was PBS 3% milk. Anti-Aβ1-42 IgG antibodies from 6E10 IgG clone (Covance), and sera from mice immunized with Aβ1-6_A2V_TAT(D) or with Aβ1-6_A2V_ emulsified in Complete Freund’s adjuvant were used as positive controls.

### Statistical analysis

Mann Withney U-test was used to compare data obtained by NOR test (Discrimination Index, Preference Index, Recognition Index). Comparison of cell viability after administration of Aβ1-42, alone or together with Aβ1-6_A2V_TAT(D) or Aβ1-6_WT_TAT(D), was performed by Student t-test. Student t-test was also used to compare (i) amyloid burden in immunohistochemical studies, (ii) Aβ40, Aβ42 and aggregated Aβ levels obtained by ELISA tests, and (iii) relative amounts of APP C-terminal fragments after Western Blot quantification. Two tailed P value equal or less than 0.05 was considered statistically significant. All calculations were performed using GraphPad Prism 5.

## Additional Information

**How to cite this article**: Di Fede, G. *et al.* Tackling amyloidogenesis in Alzheimer's disease with A2V variants of Amyloid-β. *Sci. Rep.* **6**, 20949; doi: 10.1038/srep20949 (2016).

## Supplementary Material

Supplementary Information
