# Supplementary material for: Tackling amyloidogenesis in Alzheimer’s disease with A2V variants of Amyloid-β
Source: Sci Rep. 2016 Feb 11;6:20949. doi: 10.1038/srep20949 (PMC4750079; doi:10.1038/srep20949)
Supplement: Supplementary Information [file srep20949-s1.doc]

**Supplementary Information**

**Tackling amyloidogenesis in Alzheimer’s disease with A2V variants of Amyloid-β**

Giuseppe Di Fede^1*^, Marcella Catania^1^, Emanuela Maderna^1^, Michela Morbin^1^, Fabio Moda^1^, Laura Colombo^3^, Alessandro Rossi^3^, Alfredo Cagnotto^3^, Tommaso Virgilio^1^, Luisa Palamara^1^, Margherita Ruggerone^1^, Giorgio Giaccone^1^, Ilaria Campagnani^1^, Massimo Costanza^2^, Rosetta Pedotti^2^, Matteo Salvalaglio^4^, Mario Salmona^3^, Fabrizio Tagliavini^1^

^1^Neurology V and Neuropathology Unit, IRCCS Foundation “Carlo Besta” Neurological Institute (INCB), Via Celoria 11, 20133 Milan, Italy

^2^Neuroimmunology and Neuromuscular Disorder Unit, IRCCS Foundation “C. Besta” Neurological Institute (INCB), Milan 20133, Italy.

^3^Department of Molecular Biochemistry and Pharmacology, IRCCS-Istituto di Ricerche Farmacologiche “Mario Negri”, Via La Masa 19, 20158 Milan, Italy

^4^Department of Chemical Engineering, University College London, London WC1E 7JE, UK

**Materials and Methods**

**Peptide synthesis**

All the synthetic peptides were prepared using solid-phase Fmoc chemistry on an Applied Biosystems 433A peptide synthesizer, characterized by matrix-assisted laser desorption/ionization mass spectrometry (MALDI) and their purity was always above 95 %.

Synthetic peptides Aβ1-42 in the wild-type or mutated (*H-Asp-****ALA/VAL****-Glu-Phe-Arg-His-Asp-Ser-Gly-Tyr-Glu-Val-His-His-Gln-Lys-Val-Phe-Phe-Ala-Glu-Asp-Val-Gly-Ala-Ile-Ile-Gly-Leu-Met-Val-Gly-Gly-Val-Val—OH)* form were prepared as previously described ^1^.

Aβ1-6_WT/A2V_(D) as well as the deuterated D-form analogue (^2^HDAβ1-6_WT/A2V_ in Supplementary Fig. 1S) used Fmoc amino acid D (Anaspec) on Novasyn-TGA resin^2^. The isotopically labelled peptide is composed of D-amino acids and has all Gly amino acids deuterated with a molecular weight of 2,418 Da, a mass increase of 10 Da compared to the native analogue.

Synthesis of synthetic peptides with biotin conjugate in the C-terminal position was carried out using Fmoc amino acid D (Anaspec) on Fmoc-Biotin-PEG NovaTag resin (Novabiochem). This resin incorporates a PEG spacer between the peptide and biotin moiety to reduce the possible steric hindrance. The resin can be used directly in an automated synthesizer, and the Fmoc group is removed with 20 % piperidine using standard protocols. In particular, the sequences obtained with this resin were: *i*) the sequences corresponding to residues 1-6 of the Aβ sequence in the wild-type or mutated form *(H-Asp-****ALA/VAL****-Glu-Phe-Arg-His-OH*, Biot-DAβ1-6_WT/A2V_); *ii*) the sequence corresponding to residues 48-57 of TAT protein (*Gly-Arg-Lys-Lys-Arg-Arg-Gln-Arg-Arg-Arg*, Biot-TAT) and *iii*) the sequences corresponding to residues 1-6 of the Aβ sequence in the wild-type or mutated form with a spacer of four glycines in a unique sequence with corresponding residues 48-57 of TAT protein (*H-Asp-****ALA/VAL****-Glu-Phe-Arg-His*-**Gly-Gly-Gly-Gly**-*Gly-Arg-Lys-Lys-Arg-Arg-Gln-Arg-Arg-Arg*, Biot-DTAT-Aβ1-6_WT/A2V_). All sequences were purified via reverse phase HPLC on a semipreparative C18 column (Vydac) with a mobile phase of 0.1 % TFA in water (eluent A) and 0.8 % TFA in acetonitrile (eluent B), using a linear gradient from 5 to 100 % of eluent B in 60 min.

**Resistance to protease digestion and stability in mouse serum**

The stability of Aβ1-6_A2V_TAT(D) peptide to protease digestion was also investigated. To this end, the peptide (1µg/µl) was incubated at 37 °C with 10 ng/µl of proteinase K and in the presence of OP50 *E. coli*. In fact, although the peptides contained D-amino acids, some bacterial proteolytic enzymes are able to recognize the D configuration ^3^. Samples were potted on a metallic plate (MTP 384 Ground Steel, Bruker) before and 24 h after incubation at 25 °C and 37 °C, and analyzed by MALDI TOF mass spectrometry (MS). The results indicate that Aβ1-6_A2V_TAT(D) was largely resistant to digestion with proteinase K and bacteria proteases, although a small fraction was cleaved at Gly 11 leading to formation of a peptide homologous to residues 48-57 of TAT.

Serum obtained from mouse blood was pre-warmed at 37 °C for 5 min. Aβ1-6_A2V_TAT(D) and Aβ1-6_A2V_TAT(L) peptides were dissolved in saline and added to the serum at 50 µM final concentration. The mixture was kept at 37 °C for the duration of the experiment and at different intervals an aliquot was withdrawn from the mixture, diluted 1:2 with a solution of acetonitrile:water (50:50) and agitated for 10 min. Samples were than centrifuged (4000 rpm, 5 min) and the supernatant collected for mass spectrometry analysis using MALDI-TOF instrument (Bruker Daltonics, MA, USA). For MALDI-TOF analysis, 1 µl of sample at different times was mixed with 1 µl of HCCA matrix (α-cyano-4-hydroxycinnamic acid, SIGMA ALDRICH, saturated solution in 50/50/0.1 % water/acetonitrile/trifluoroacetic acid) and spotted on a MALDI target plate. Mass spectra were acquired in reflector mode and elaborated using the Flex Analysis program.

**Aβ1-6_A2V_TAT(D) levels in mouse brain**

To verify Aβ1-6_A2V_TAT(D) distribution *in vivo*, and particularly its bioavailability in the brain, we determined Aβ1-6_A2V_TAT(D) levels in mice brains after i.p. injection at a dose of 10 mg/kg. Mice were sacrificed at two intervals (1 h, 24 h). Aβ1-6_A2V_TAT(D) was then quantified in mouse brain using HPLC-MS/MS.

The quantitative levels of Aβ1-6_A2V_TAT(D) in the brain were determined by following a previously described procedure ^4^.

*Preparation of standards and calibration curve.* HPLC-grade acetonitrile, formic acid (98 %) and isopropanol were purchased from Fluka (Buchs, Switzerland). HPLC grade MilliQ water was obtained with a MILLI-RO PLUS 90 apparatus (Millipore, Molsheim, France). Primary stock solutions of Aβ1-6_A2V_TAT(D) and the deuterated analogue (internal standard (IS)) were separately prepared with the mobile phase (HCOOH 0.1 % + ACN 1 %) at a concentration of 20 µM. Then primary stock solutions were diluted with the mobile phase to prepare standard working solutions of Aβ1-6_A2V_TAT(D) and IS. The standard calibration curves were prepared in untreated tissue extract (brain slices) in six different concentrations of Aβ1-6_A2V_TAT(D) (0-30 ng), all containing IS at 20 ng and were freshly prepared for every analysis batch. Standard samples for check stability of Aβ1-6_A2V_TAT(D) were prepared at 5, 15 and 30 ng, all with 20 ng of IS in untreated tissue extract.

*Sample preparation (extraction).* Tissue samples (50 mg brain slices) were homogenized with 200 μL of 50 % acetonitrile, 5 % TFA in water with a handbook pestle for microcentrifuge tube, were stirred for 10 minutes at 4 °C and centrifuged at 3000 rpm for 5 min at 4 °C. The supernatant was then collected and the pellet was re-extracted with 100 ul of solution, stirred for 10 minutes at 4 °C and centrifuged at 6,000 rpm for 5 minutes at 4 °C. The supernatant was collected and the cycles of re-extraction, stirring and centrifugation were repeated one more time. At last all the supernatant was collected, centrifuged at 12,000 rpm for 30 seconds (at 4 °C) and lyophilized on Heto HSC 500 (Thermo Fisher Scientific, MA, USA). After lyophilisation, samples were stocked at -20 °C before HPLC-MS/MS analysis. Just before the analysis, the samples were suspended in 100 μL of 0.1 % HCOOH in water + 1 % acetonitrile in auto-sampler vials. The IS (20 ng) was added to the treated sample before homogenization. For the calibration curves the STD (0-30ng) and IS (20 ng) were added when the sample was suspended in 0.1 % HCOOH in water + 1 % acetonitrile in auto-sampler vials for analysis in HPLC-MS/MS.

*Liquid chromatography (HPLC) and tandem mass spectrometry (MS-MS).* High pressure liquid chromatography tandem mass spectrometry (HPLC-MS/MS) analysis was performed using a system consisting of a 200 series pump and autosampler (Perkin Elmer), interfaced to an API 3000 triple quadrupole mass spectrometer, equipped with a turbo ion spray source (AB Sciex, Toronto, Canada). The ANALYST 1.5.1 software package was used for data collection and processing (AB SCIEX). The analyte Aβ1-6_A2V_TAT(D) and its IS were separated at room temperature by injecting 20 μL of extracted sample onto a Jupiter C4 300 A analytical column, 2 x 150mm, 5µm particle size (Phenomenex). A gradient elution was used for chromatographic separation, using 0.1 % formic acid in water as solvent A, and acetonitrile + 20 % isopropanol as solvent B at a flow rate of 150 µl/min. The elution started with 99 % of eluent A and 1 % of eluent B maintained for 2 min, followed by a 5 min linear gradient to 50 % of eluent B, a 1 min linear gradient to 99 % of eluent B, a 2 min isocratic elution and a 0.5 min linear gradient to 1% of eluent B, which was maintained for 12.5 min to equilibrate the column. The samples were maintained at 4 °C in the autosampler. The mobile phase was directly introduced into the ion source, which operated with a turbo ion gas at 500 °C and ion spray voltage of 5,000 V. Peptides were then detected on a Perkin Elmer series 200 QQQ mass spectrometer using the following parameters: positive ion mode, using zero air as nebulizer gas (35 psi) and as heater gas (50 psi). Gas flow rate 8 L/min at 14.7 PSI at 70 °F, nitrogen was employed as curtain gas (20 psi) and as collision gas at 5 psi. And Q1 and Q3 set to unit resolution.

*Peptide recovery from mouse brain.* Sample A: untreated tissue (50 mg brain mouse APP23 slices) was homogenized with 200 μL of 50 % acetonitrile, 5 % TFA in water with 20ng of Aβ1-6_A2V_TAT(D) with a handbook pestle for microcentrifuge tube, and repeated passages of extraction as described in “sample preparation”. Just before the analysis, the samples were suspended in 100 μL of 0.1 % HCOOH in water + 1 % acetonitrile with 20 ng of IS in auto-sampler vials. Sample B: untreated tissue (50 mg brain mouse APP23 slices) was homogenized with 200 μL of 50 % acetonitrile, 5 % TFA in water with a handbook pestle for microcentrifuge tube, and repeated passages of extraction as described in “sample preparation”. Just before the analysis the samples were suspended in 100 μL of 0.1 % HCOOH in water + 1 % acetonitrile with 20 ng of IS and 20ng of Aβ1-6_A2V_TAT(D) in auto-sampler vials.

**Results**

**Characterization of Aβ1-6_A2V_TAT(D) and Internal Standard (IS)**

The full mass spectra of Aβ1-6_A2V_TAT(D) and IS (at 5 ng/μL) in 50 % ACN / HCOOH 0.1 % were acquired in positive ESI mode. As is typical of ESI mass spectra, the mass spectrum of native Aβ 1-6_A2V_TAT (D) showed multiply charged ions, with two main peaks at *m/z* 482.9 and 402.6 that are the species corresponding to the M+5H^+^ and M+6H^+^ ions, respectively. The mass spectrum of the deuterated analogue exhibited two major peaks at m/z 485.0 and 404.3, corresponding to the equivalent multiple-charged ions of native Aβ1-6_A2V_TAT(D).

After optimizing focusing potential (FP), declustering potential (DP) and collision energy (CE), the precursor/product ion pairs at *m/z* 402.6/440.2 and *m/z* 404.3/442.0 were selected in the SRM mode (measuring the fragmentation products of the multiply charged protonated pseudo-molecular ions) for quantification of Aβ1-6_A2V_TAT(D) and IS, respectively. In addition to the most abundant ions from Aβ1-6_A2V_TAT(D) and IS, three other precursor/product ion pairs were obtained for Aβ1-6_A2V_TAT(D) and IS and were selected as confirmatory ions for the identification of Aβ1-6_A2V_TAT(D) and IS in samples. Quantitative analysis was performed by monitoring multiple reactions, as summarized in Supplementary Table 1S.

**Method Validation: linearity and lower limit of quantification**

Calibration curves from 1 ng to 30 ng (2.5 to 250 fmol/μL) were obtained by plotting the peak area ratio of Aβ1-6_A2V_TAT(D) to IS against the corresponding spiked concentration. A representative calibration curve is shown in Supplementary Figure 2S, and the linear regression coefficients (R^2^) were above 0.99, showing that this method is linear in this concentration range.

The lower limit of detection (LLOD, assuming a 3-fold signal to noise ratio) of the assay was 150 pg injected. The lower limit of quantification (LLOQ assuming a 10-fold signal to noise ratio) was 500 pg injected. Calibration curves were prepared in untreated tissue extract (brain) to confirm the linearity of the method.

**Storage stability and precision**

Intra- and inter-run precision of the assay was checked with standard samples (prepared in untreated mouse brain) spiked with Aβ1-6_A2V_TAT(D) at three concentration levels (5, 10 and 30 ng with 20 ng of IS), during different days (Supplementary Table 2S). The average coefficient of variation (CV) was 1.26 % and 1.18 % for intra- and inter-assay precision, respectively.

**Serum stability and recovery of peptide from mouse brain**

MALDI-TOF analysis showed that Aβ1-6_A2V_TAT(D) remained stable for 48 h, while Aβ1-6_A2V_TAT(L) was rapidly degraded in about 1 h (Supplementary Fig. 3S).

The peptide recovery in untreated tissue was 80.37 % (Supplementary Table 3S and Fig. 4S). This recovery percentage was determined by analysis in triplicate for each sample type and demonstrated the validity of extraction method.


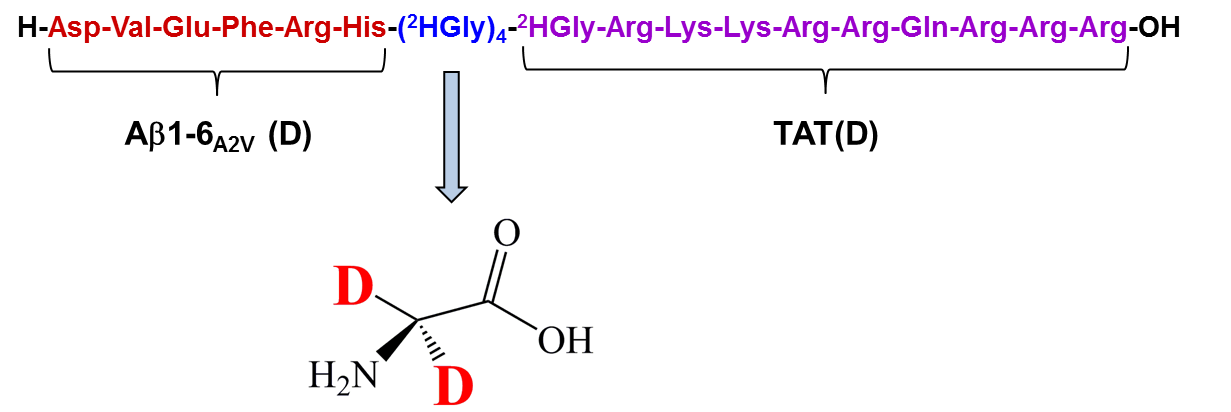


**Supplementary Figure 1S Structure of the deuterated Aβ1-6_A2V_TAT(D) *(^2^HDAβ1-6_A2V_).***

All amino acids are enantiomers in the D form. Deuterated amino acids are indicated with the **^2^**H first amino acid code. The molecular weight of each glycine is 77 Da. A total of 10 deuterium atoms are present in the standard.


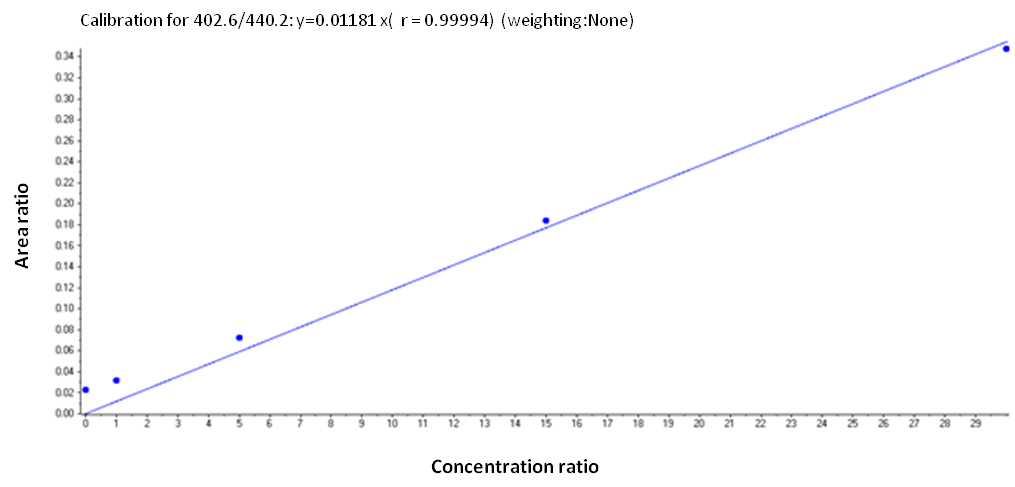


**Supplementary Figure 2S Calibration Curve, Linearity representative of the method.** Quantitative HPLC-MS/MS analysis was performed in the 0-30 ng Aβ1-6_A2V_TAT(D) concentration range and plotted vs the ratio of Aβ1-6_A2V_TAT(D)/Aβ1-6_A2V_TAT(D) D10.

**
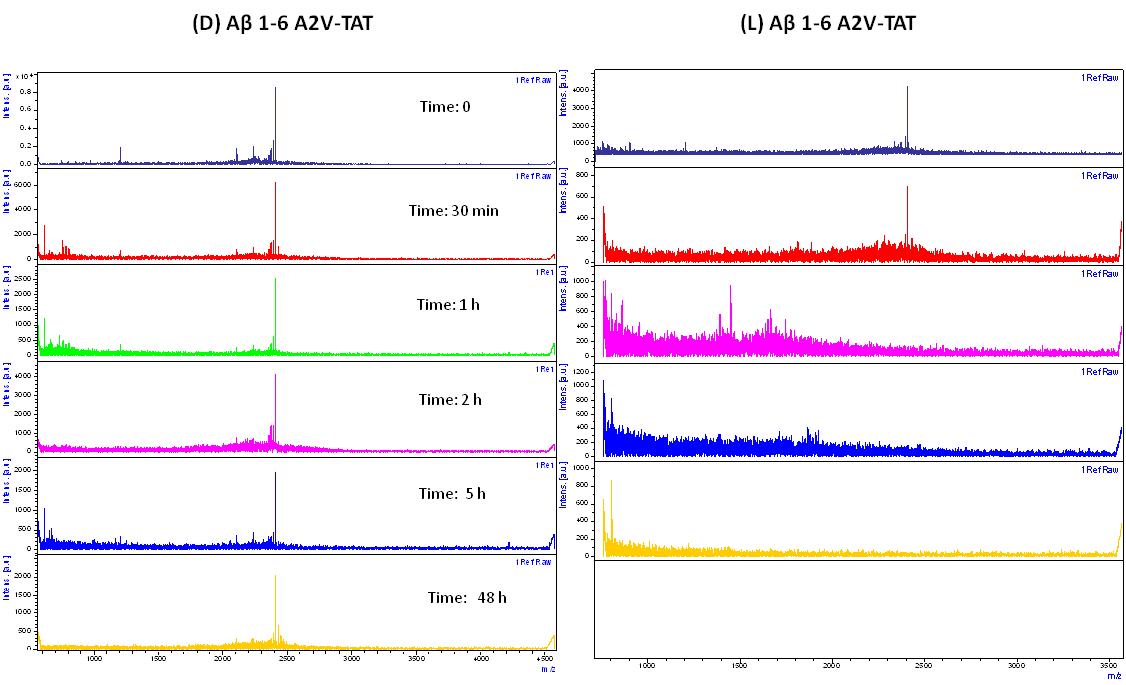
**

**Supplementary Figure 3S Stability of Aβ1-6 A2V-TAT(D) and (L) peptides in human serum.** Peptides (50 µM) were incubated with human serum at 37 °C. At different times after incubation (from 0 up to 48h), aliquots of the samples were spotted on a metallic plate (MTP 384 Ground Steel, Bruker) and analyzed via MALDI TOF mass spectrometry.


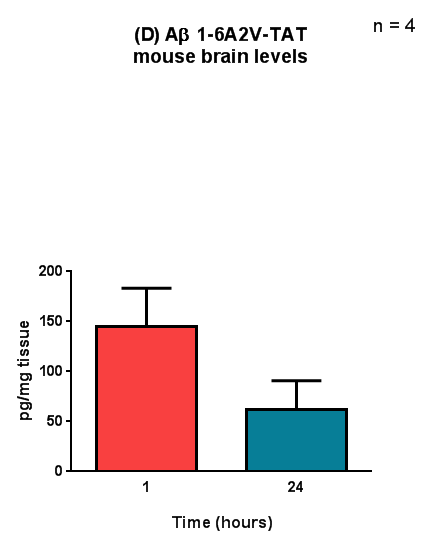


**Supplementary Figure 4S** **Aβ1-6_A2V_TAT(D) levels in mouse brain (n=4).** Data are reported as mean ± SEM of peptide/mg tissue. Animals were treated i.p. at a dose of 10 mg/kg. Animals were sacrificed 1 h or 24 h after last administration following 21 weekly treatments (**p* < 0.05 T1-1h vs T1-24h and Student’s *t*-test).

**Supplementary Table 1S.** Main parameters used for multiple reactions monitoring quantitative analysis

| **Analyte** | **Precursor / Product Ion** | **Focusing Potential (V)** | **Collision Energy (eV)** |
| --- | --- | --- | --- |
| Aβ1-6_A2V_TAT(D) | 402.6 / 440.2 (quantifier) | 140 | 18 |
|  | 402.6 / 436.6 (qualifier) | 140 | 20 |
|  | 482.9 / 550 | 180 | 24 |
| Aβ1-6_A2V_TAT(D) D10 | 404.3 / 442 (quantifier) | 140 | 18 |
|  | 404.3 / 438.4 (qualifier) | 140 | 20 |
|  | 485 / 552.5 | 180 | 24 |

**Supplementary Table 2S**. Inter- and intra-day precision data for the developed method

|  | **Interday** |  | **Intraday** |  |
| --- | --- | --- | --- | --- |
| ng | SD | CV % | SD | CV % |
| 5 | 0.11 | 1.73 | 0.05 | 0.82 |
| 15 | 0.22 | 1.44 | 0.35 | 2.35 |
| 30 | 0.11 | 0.38 | 0.18 | 0.61 |

**Supplementary Table 3S.** Recovery of Aβ1-6_A2V_TAT(D) from untreated tissue

| **First injection** | | | | | **Second injection** | | | |
| --- | --- | --- | --- | --- | --- | --- | --- | --- |
|  | **Average 3 samples (ng)** | **St. Dev.** | **C.V. (%)** | **Recovery (%)** | **Average 3 samples (ng)** | **St. Dev.** | **C.V.**  **(%)** | **Recovery (%)** |
| **Sample A** | 16.34 | 2.30 | 14.08 | 81.70 | 15.81 | 2.78 | 17.61 | 79.03 |
| **Sample B** | 19.54 | 0.94 | 4.83 | 97.70 | 20.22 | 4.43 | 21.93 | 101.12 |
| **Average % Recovery**  **80.37** | | | | | | | | |

**References**

1 Manzoni, C. *et al.* Overcoming synthetic Abeta peptide aging: a new approach to an age-old problem. *Amyloid* **16**, 71-80, doi:10.1080/13506120902879848 (2009).

2 Di Fede, G. *et al.* A recessive mutation in the APP gene with dominant-negative effect on amyloidogenesis. *Science* **323**, 1473-1477, doi:10.1126/science.1168979 (2009).

3 Asano, Y. & Lübbehüsen, T. L. Enzymes acting on peptides containing D-amino acid. *J Biosci Bioeng* **89**, 295-306 (2000).

4 Davoli, E. *et al.* Determination of tissue levels of a neuroprotectant drug: the cell permeable JNK inhibitor peptide. *J Pharmacol Toxicol Methods* **70**, 55-61, doi:10.1016/j.vascn.2014.04.001 (2014).
